# Reanalysis of ProteomicsDB Using an Accurate, Sensitive, and Scalable False Discovery Rate Estimation Approach for Protein Groups

**DOI:** 10.1016/j.mcpro.2022.100437

**Published:** 2022-11-01

**Authors:** Matthew The, Patroklos Samaras, Bernhard Kuster, Mathias Wilhelm

**Affiliations:** 1 Chair of Proteomics and Bioanalytics, Technical University of Munich (TUM), Freising, Germany; 2 Bavarian Biomolecular Mass Spectrometry Center (BayBioMS), Technical University of Munich (TUM), Freising, Germany; 3 Computational Mass Spectrometry, Technical University of Munich (TUM), Freising, Germany

**Keywords:** large-scale proteomics, protein false discovery rate estimation, picked protein FDR, protein inference, ProteomicsDB, bpP, best percolator PEP, cT, classic TDS, dS, discarded shared peptides, FDR, false discovery rate, mmP, multiplication of MaxQuant PEPs, MS, mass spectrometry, PEP, posterior error probability, pgT, picked group TDS, PSM, peptide-spectrum match, pT, picked TDS, rS, razor peptide, rsG, rescued subset protein grouping, sG, subset protein grouping, TDS, target-decoy strategy

## Abstract

Estimating false discovery rates (FDRs) of protein identification continues to be an important topic in mass spectrometry–based proteomics, particularly when analyzing very large datasets. One performant method for this purpose is the Picked Protein FDR approach which is based on a target-decoy competition strategy on the protein level that ensures that FDRs scale to large datasets. Here, we present an extension to this method that can also deal with protein groups, that is, proteins that share common peptides such as protein isoforms of the same gene. To obtain well-calibrated FDR estimates that preserve protein identification sensitivity, we introduce two novel ideas. First, the picked group target-decoy and second, the rescued subset grouping strategies. Using entrapment searches and simulated data for validation, we demonstrate that the new Picked Protein Group FDR method produces accurate protein group-level FDR estimates regardless of the size of the data set. The validation analysis also uncovered that applying the commonly used Occam’s razor principle leads to anticonservative FDR estimates for large datasets. This is not the case for the Picked Protein Group FDR method. Reanalysis of deep proteomes of 29 human tissues showed that the new method identified up to 4% more protein groups than MaxQuant. Applying the method to the reanalysis of the entire human section of ProteomicsDB led to the identification of 18,000 protein groups at 1% protein group-level FDR. The analysis also showed that about 1250 genes were represented by ≥2 identified protein groups. To make the method accessible to the proteomics community, we provide a software tool including a graphical user interface that enables merging results from multiple MaxQuant searches into a single list of identified and quantified protein groups.

## Highlights

• Evaluating protein group FDR estimation methods with entrapment and simulated data.
• Accurate & sensitive protein group FDR method on databases with protein isoforms.
• Tool for combining multiple large-scale MaxQuant searches on protein group-level.
• Analysis on ProteomicsDB identified >1200 human genes with multiple protein groups.

## Graphical Abstract

**Figure:**
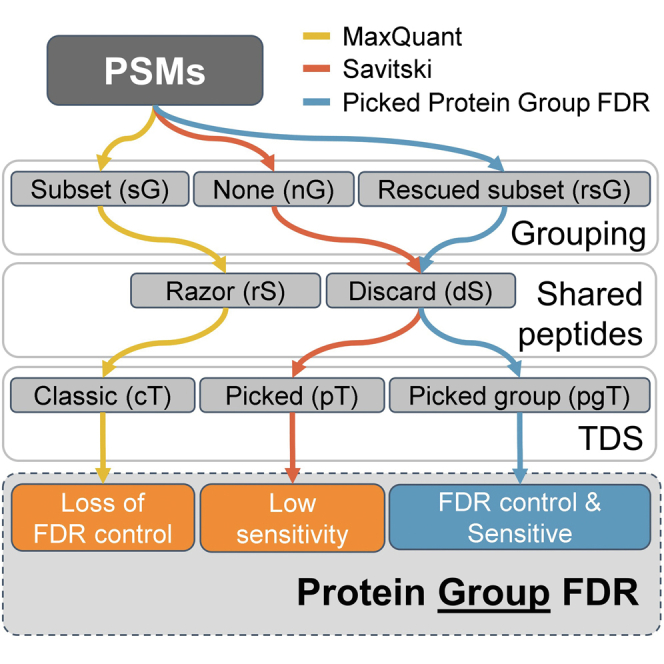

## In Brief

Distinguishing between protein products of a gene is complicated by many peptides that such isoforms have in common. Grouping indistinguishable proteins alleviates this issue but leads to problems with estimating false discovery rates (FDRs) in large-scale experiments as false positives accumulate. Here, protein group FDR estimation methods were evaluated on accuracy and sensitivity. Our new Picked Protein Group FDR method performed best and reanalysis of the draft human proteome in ProteomicsDB found >1200 genes with multiple protein products.

Algorithms for protein quantification and identification from mass spectrometry (MS) data are continuously challenged by the ever growing trend towards large-scale experiments. Today, it is not at all uncommon to perform experiments resulting in hundreds, or even thousands, of MS data files (1, 2, 3, 4). While such data is increasingly deposited into searchable public data repositories such as ProteomicsDB (5), PRIDE (6), MassIVE (http://massive.ucsd.edu), or PeptideAtlas (7), estimating the proportion of false positive identifications (false discovery rate; FDR) at the gene-level is a nontrivial task (8, 9, 10). An even larger challenge is presented by distinguishing between different protein products from the same gene (11), such as splice variants (12) or SNPs or when analyzing mixtures of orthologous proteins from different species such as human/mouse xenografts or bacterial communities in metaproteomics (13).

The most prevailing method for estimating FDRs makes use of so-called target-decoy models (14), where decoy peptide/protein sequences are added to the collection of genuine (*i.e.*, target) peptide/protein sequences to serve as a model for false hits. The underlying assumption is that the database search engine identification score distributions of decoy and incorrect target peptide-spectrum matches (PSMs), peptides, and proteins are the same. Violations of this assumption can lead to inaccurate FDR estimates, referred to as loss of FDR control. A well-working FDR estimation procedure should both be accurate, that is, reflecting the true proportion of false discoveries, and sensitive, that is, maximizing the number of acceptable discoveries at a defined threshold (typically 1%). When large-scale datasets begun to appear in the literature, it was soon recognized that naively compiling lists of identified proteins by combining large numbers of experiments led to loss of control of the protein FDR (15, 16) and that applying the simple target-decoy approach led to issues with reduced sensitivity (1). The latter turned out be the result of an unintended asymmetry between decoy proteins and falsely identified target proteins. This is because false target PSMs may arise from both correct and false target proteins, whereas decoy PSMs only arise from decoy proteins, which are false by definition (17).

This asymmetry was subsequently resolved by the development of the picked target-decoy strategy (picked TDS) (8). Briefly, the picked TDS (pT) compares the highest observed search engine PSM score for a target protein with its respective sequence-reversed or shuffled decoy protein and only retains the entry with the highest score. This strategy re-established the assumption that false target protein identifications have the same score distribution as decoy protein identifications. As a result, the pT increased the number of confidently identified proteins compared to the classic TDS (8, 9). Savitski *et al.* (8) employed this pT to calculate protein-level FDRs using a method called the Picked Protein FDR approach. This method was designed to operate at the gene level and, for simplicity, discarded identified peptides that are shared between multiple protein sequences. This choice was made because shared peptides are rare when only canonical protein sequences are considered. However, not considering shared peptides led to reduced sensitivity when searching databases containing multiple protein isoforms of a gene. This is because if a peptide is shared between two protein isoforms of a gene but is the only peptide identified for that gene, the pT would discard this peptide and neither isoform of the gene would be reported.

As proteomic technology has improved over time to enable profiling proteomes at more and more depth, the above simplification may no longer be acceptable in many circumstances. Indeed, it has been estimated that up to 50% of all peptide sequences are shared between multiple protein sequences when considering all protein isoforms resulting from alternative RNA splicing (18). In turn, this leads to complications for protein identification and quantification (19). If a peptide is shared between two protein isoforms of a gene and if it is the only peptide identified for that gene, one can confidently mark the gene as identified but one cannot be certain regarding which of the two isoforms (or both) are identified. A popular way to address this issue is to combine proteins that share identified peptides into a group and treat such a group of proteins as a single entity (20). In the above simple example, the two protein isoforms would be combined and treated as one protein group with the interpretation that at least one of the two sequences was actually present in the sample (21). In reality, both shared and distinct (*i.e.*, unique) peptides may be identified for protein isoforms of the same gene. Therefore, proteins are typically only grouped if a protein’s identified peptides are a subset of the identified peptides of another protein. The identification of even a single unique peptide for each of the isoforms would prevent isoforms to be grouped even if they had (many) identified peptides in common. This may lead to the identification of multiple protein groups for a given gene and, in some cases, even particular protein isoforms.

Protein grouping has become very popular because it provides some of the desired granularity for distinguishing different protein products from the same gene. However, the subject of estimating protein group-level FDR has received little attention (20, 21, 22). In particular, three unresolved problems may be noted. First, the pT cannot directly be applied to protein groups, as the decoy counterparts of the target proteins in a protein group are not necessarily also grouped together. This is because the decoy counterpart proteins have their own set of identified shared and unique (decoy) peptides upon which grouping is based. One could artificially group all decoy counterparts into a decoy protein group but this violates the need to treat target and decoy proteins in the same manner, which is at the very heart of the target-decoy idea. Second, protein grouping leads to a practical problem when combining or comparing large datasets. Because the composition of protein groups depends on the set of identified peptides, proteins that were grouped together in one dataset are likely not grouped with the same composition of proteins in another dataset. This makes comparisons between datasets difficult unless all the data is combined and searched again, which is not practical for very large datasets or when looking at entire repositories. Recently, a tool to produce protein grouping results for multiple Percolator output files was released designed for metaproteomic datasets (23). However, to the best of our knowledge, no tools exist yet that can easily combine results from multiple MaxQuant searches with consistent protein grouping. Third, even with protein grouping, many high-confident peptides may still be shared between multiple protein groups and can, therefore, not unequivocally be attributed to a single protein group. Some methods apportion such cases over the protein groups concerned using probabilistic models (24, 25, 26, 27) but these often require the data to adhere to specific probability distributions, have problems with scaling, and have hard-to-interpret models and results (28). To avoid such complications, a popular alternative is the Occam's razor heuristic (also known as the "law of parsimony" or “razor peptides” (rS)), most notably employed in the MaxQuant software platform (29). Here, a typical decision rule for shared peptides is to assign them to the protein group with the highest number of identified unique peptides and arbitrarily pick one of the protein groups in case of a tie. Although such rules may pick the correct protein group in a majority of cases, the number of cases where it picks the wrong protein group accumulates in large-scale experiments. Worse still, these cases do not arise from decoy protein groups because, whichever decoy protein group a shared decoy peptide is attributed to, is false by definition. Therefore, these false positive targets remain unaccounted for when computing FDRs, leading to a loss of FDR control and anticonservative FDR estimates (28).

Note that gene-level FDRs and protein group-level FDRs differ fundamentally due to the type of entities in the lists they are calculated on, that is, genes and protein groups respectively and can, thus, generally not be compared to each other. However, one can calculate gene-level FDRs on a list of protein groups by, for example, only retaining the best scoring protein group per gene. The reverse, that is, calculating protein group-level FDRs from a list of genes, is only possible in the most trivial sense, where each gene is its own protein group. In the following, we will exclusively use protein group-level FDRs.

To address the above issues, we introduce the Picked Protein Group FDR method, along with an accompanying software tool, which extends the Picked Protein FDR approach to protein groups. In particular, we show that this method identifies up to 4% more protein groups than the method used by MaxQuant while simultaneously correctly controlling the protein group-level FDR. Furthermore, we show that the method scales to very large numbers of experiments, exemplified by the reanalysis of the entire human section of ProteomicsDB. The tool comes with a graphical user interface operating under Windows as well as a Python package. The software also contains the option to merge results from multiple searches into a single list of quantified protein groups (label-free quantification, intensity based absolute quantification, tandem mass tag) and provides an output similar to MaxQuant’s proteinGroups.txt. Instructions for download and use can be found at https://github.com/kusterlab/picked_group_fdr.

## Experimental Procedures

### Datasets

RAW files for a deep proteome study of 29 healthy human tissues (Wang *et al.* (30), PXD010154; HCD data only) corresponding to 50 million MS2 spectra were searched using MaxQuant (v1.5.3.8) against the human Swiss-Prot database including isoforms and TrEMBL entries downloaded from UniProt (accessed: March 27, 2020, 95,943 protein sequences), concatenated with a list of contaminants, as provided with MaxQuant, as well as reversed decoy sequences for all entries. Trypsin was specified as protease with up to two missed cleavages. Both peptide and protein-level FDR thresholds were set to 100% and default values were used for all other parameters. This data set is referred to in this article as the *Wang_base* dataset.

All PSMs assigned to human samples in ProteomicsDB (accessed: June 08, 2020) including both Mascot and MaxQuant search results for all proteases formed the *PrDB* dataset used in the current study and consists of 77 distinct projects of varying size (supplemental Table S1), totaling 269 million target and 141 million decoy PSMs (100% PSM-level FDR, searched against Swiss-Prot database including isoforms and TrEMBL entries).

For both the *Wang_base* and *PrDB* datasets, we produced three lists of PSMs that were filtered to only include PSMs with peptide sequences that mapped to the canonical Swiss-Prot, the Swiss-Prot with isoforms databases and the Swiss-Prot+TrEMBL with isoforms, respectively. These protein sequences were obtained from ProteomicsDB (accessed: July 05, 2020). Note that these filtered lists of PSMs will slightly differ from the results of a normal search against these respective databases. In the filtered lists, PSMs with sequences not present in the reduced databases are discarded instead of matched to another sequence within the reduced database. As this equally affects incorrect target and decoy PSMs, this should not lead to any biases for FDR estimation.

### Entrapment Searches

To assess if protein group-level FDRs are well-calibrated for the protein group-level FDR estimation methods presented below, entrapment searches were performed (31). Briefly, in entrapment searches, the target database is extended by an entrapment database, typically 5 to 10 times the size of the target database, which only contains protein sequences known to be false.

Entrapment databases were constructed as follows (for a graphical overview, see supplemental Fig S1*A*):

1. The target database was in-silico digested with Trypsin/P as protease, without missed cleavages. Only peptides longer than six amino acids were retained.
2. A fraction *S* (see below) of peptides was randomly selected to remain unchanged, thereby creating shared peptides between the target and entrapment databases (9).
3. All other peptide sequences were shuffled, while keeping the C-terminal amino acid the same.
4. Finally, these shuffled peptides replaced their original versions in the target protein sequence, resulting in an entrapment protein having the same number of shared peptides as the target version.
5. Steps 2 to 4 were repeated four times to yield an entrapment database five-times the size of the original target database.

Entrapment databases were generated for two different fractions *S* of shared peptides, with *S* = 0.5 representing the shared ratio of the Swiss-Prot+Isoforms database and *S* = 0.04 representing the shared ratio of the Swiss-Prot database. The spectra from *Wang_base* were searched against each of these entrapment databases with MaxQuant (v1.5.3.8). Trypsin/P was specified as protease and no missed cleavages were allowed, to reflect the construction of the entrapment databases. The peptide-level FDR threshold was set to 10% (searching with 100% peptide-level FDR was prohibitively slow) and the protein-level FDR threshold to 100%. All other parameters were set to default values. These datasets are referred to *Wang_trap_0.5* and *Wang_trap_0.04*, respectively.

### Peptide-Level Filtering

For protein-level FDR estimation methods, it is common to supply the list of PSMs without applying a peptide-level FDR threshold (8, 9). This allows the estimation of protein-level FDRs for proteins with only weak evidence. This could be especially relevant for distinguishing protein isoforms, which usually have few unique peptides. Note that, for parsimony-based protein inference methods that do not apply protein-level FDR estimation, it is vital to apply a strict peptide-level FDR threshold before protein grouping to prevent excessive accumulation of false protein groups.

However, for MaxQuant’s method, we noted a decrease of up to 9% in the number of identified protein groups at 1% protein group-level FDR for the 100% peptide-level FDR cutoff compared to MaxQuant’s default 1% peptide-level FDR cutoff (supplemental Fig. S2). This decrease was due to the addition of low confident peptides that were filtered out in the 1% peptide-level FDR cutoff results. As MaxQuant computes protein group scores by a multiplication of posterior error probabilities (PEPs), these low confident peptides actually decreased the confidence in their corresponding protein relative to proteins without low confident peptides. To make the comparison to MaxQuant fairer and to rule out the permissive FDR threshold as a confounding factor when comparing methods, we applied a 1% peptide-level FDR cutoff for all methods. This cutoff was applied per raw file, as is done in MaxQuant.

### Percolator PSM Rescoring

For the three *Wang* datasets, the *evidence.txt* MaxQuant output files were processed by a custom python script to create a percolator input file by extracting the following features: Andromeda score, Andromeda delta score, peptide length, charge (one-hot encoded), mass, enzymatic N-terminal, enzymatic C-terminal, missed cleavages, number of modifications, delta mass, and absolute delta mass. This file was subsequently processed by Percolator v3.04. The resulting PSM target and decoy output files were merged back with the original *evidence.txt* and the Q-value and PEP columns were updated with the new values provided by the Percolator analysis.

For the *PrDB* dataset, PSMs were grouped by project (n = 77) and the same features as above were extracted, minus the delta mass and absolute delta mass features. These two columns were incomplete in ProteomicsDB and were, therefore, discarded. Percolator was applied to each project separately, so that the weights of the support vector machine could be adjusted for each project. The percolator results were extracted on peptide level, where only the best scoring PSM per peptide sequence was retained. The resulting peptide lists (one for each project) were merged into a single list, using the -log10(q-value) of the peptide as the score and only retaining the best PSM for each peptide. Peptide-level q-values were then recalculated on this final list of (unique) peptide sequences.

### Simulated Data

Entrapment searches provide a good way to assess the validity of FDR estimates, but as it requires researching the data, this becomes prohibitively computationally expensive for datasets comprising hundreds of experiments. Therefore, lists of peptides were simulated with respective scores and labels (correct/incorrect) that recapitulate experimental results to a degree that they agree with the observations made with entrapment searches on a qualitative level. Specifically, the *proteotypicity* was used to select peptides, that is, conditional probabilities of detecting a peptide, given that the corresponding protein was identified. Furthermore, the probabilities of a protein to be detected were used, to replicate the typical observation that some proteins are identified in almost every experiment, whereas others might only be identified in only one or a few of experiments. Both the proteotypicity values (*protein_probs*) and protein detection probabilities (*peptide_probs*) were calculated from the data in ProteomicsDB. The simulation is described in pseudo-code below.

**Table:** Pseudocode for generation of simulated datasets

Input:- *n_exp*: number of experiments- *n_prot_mean*: mean number of proteins present per experiment- *n_prot_stdev*: stdev number of proteins present per experiment- *tp_score_mean*: mean of score distribution for true positives- *tp_score_stdev*: stdev of score distribution for true positives- *fp_score_mean*: mean of score distribution for false positives- *fp_score_stdev*: stdev of score distribution for false positives- *incorrect_ratio*: proportion of incorrect peptides without FDR threshold- *peptide_fdr*: peptide fdr threshold, should be lower than protein_fdr- *protein_probs*: probability for each protein to be present- *peptide_probs*: probability for each peptide (including shared peptides!) to be present given that the protein is present Algorithm: 1. For each experiment a. Pick *n_prot* ∼ *N(n_prot_mean, n_prot_stdev)* proteins to be present b. Draw *n_prot* true positive target proteins from the target database c. Calculate minimum peptide score corresponding to given *peptFDR* and *incorrect_ratio*: *min_score* = Φ^-1^(1 - *peptFDR* ∗ (1 - *incorrect_ratio*) / *incorrect_ratio;* *fp_score_mean*, *fp_score_stdev*) d. For each true positive target protein i. Draw true positive peptides based on their *peptide_probs* ii. Draw score for each true positive peptide from a truncated normal distribution: *trunc_norm*(*min_score*, ∞; *tp_score_mean*, *tp_score_stdev*) e. Randomly draw 2 ∗ *tp_peptides* ∗ *peptide_fdr* / (1 - *peptFDR*) false positve peptides from all peptides in target and decoy databases f. Draw score for each false positive peptide from a truncated normal distribution: *trunc_norm*(*min_score*, ∞; *fp_score_mean*, *fp_score_stdev*) 2. Do protein grouping based on observed peptides 3. Calculate protein group FDRs

For the simulated datasets in this article, the input parameters were estimated from the Wang_base dataset, where one experiment corresponds to one of the 29 tissues analyzed: *n_protein_mean* = 10.000, *n_protein_stdev* = 1000, *tp_score_mean* = 2.5 *tp_score_stdev* = 0.7, *fp_score_mean* = 0.0, *fp_score_stdev* = 0.7, *incorrect_ratio* = 0.6, *peptide_fdr* = 0.01. Data was simulated for 10, 100, 200, 300, 400, and 500 experiments, to assess the performance of the different protein group-level FDR estimation methods at different scales. Additionally, datasets were simulated where the combined list of experiments had a 10% peptide-level FDR per raw file or a global 1% peptide-level FDR.

### Protein Grouping

A common practice to alleviate the problem of distributing shared peptide identifications between multiple protein sequences is to group proteins into so-called protein groups. In this article, we considered the following options:

#### No Grouping

Each protein is considered its own protein group. In databases with many protein isoforms or homologous proteins, this will result in many shared peptides between the protein groups.

#### Subset Grouping

Proteins are grouped if the peptides for one protein form a subset of the peptides for a second protein. This will, for example, group isoforms of the same gene if no isoform-specific peptides are identified. This method is, for example, used by MaxQuant.

#### Rescued Subset Groupin

The idea behind this new two-step procedure is to prevent that protein groups are split into multiple groups due to the presence of low-confident PSMs. To achieve this, first, a regular subset protein grouping (sG) is performed, producing a list of protein groups, PG1. Next, we filter the list of PSMs using a PSM-level threshold equivalent to a 1% protein group-level FDR, which is calculated based on PG1. Then, a second sG is performed using this filtered list of PSMs, producing a second list of protein groups PG2. Finally, PG2 is supplemented with protein groups from PG1 that did not contain proteins already present in a protein group in PG2. This last step ensures that protein groups above 1% protein group-level FDR also have FDR estimates.

### Shared Peptides

After protein grouping, peptides have to be assigned to protein groups. This is simple if all proteins the peptides could originate from are all in the same protein group. However, one needs to decide on how to deal with peptides shared between proteins that are not in the same protein group. Two options are considered in this article:

#### Razor Peptides

The rS strategy forces the assignment of a shared peptide to one of its associated protein groups based on which of these had the highest number of unique peptides. In case of a tie for the highest number of unique peptides, the tie is broken randomly.

#### Discard Shared Peptides

Here, all peptides that are shared between protein groups are discarded.

### Protein Group Scoring

To rank protein groups by confidence of identification, protein group scores are computed based on the identification probabilities of the constituent peptides. Here, three options for protein group scoring were evaluated.

#### Multiplication of MaxQuant PEPs

The protein group score used by MaxQuant is based on multiplying peptide PEPs. This score was re-implemented in Python and verified by comparing it to the protein group score that MaxQuant itself reported for each protein group. In reproducing the protein group scores reported by MaxQuant, we noted that the peptide PEPs were first divided by a constant, which appeared to be chosen with a view to maximizing the number of identified protein groups. Therefore, we implemented a grid search to optimize this constant before calculating the protein group scores.

#### Best MaxQuant PEP

This takes the −log10(PEP) of the best scoring PSM for the protein as the protein group’s score.

#### Best Percolator PEP

This takes the −log10(PEP) of the best scoring PSM after rescoring with Percolator as the protein group’s score.

### Protein (Group) TDS

Before protein group-level FDR estimation, one can optionally perform a target-decoy competition step on the protein group-level. This competition has been shown to resolve problems with decreased sensitivity that results from an asymmetry between decoy proteins and falsely identified target proteins as noted in the introduction.

#### Classic TDS

All proteins are passed to the FDR calculation without any protein-level target-decoy competition taking place.

#### Picked TDS

This is a target-decoy competition step at the protein level. For each target protein, one notes down the corresponding decoy protein that was constructed by reversing or shuffling the target protein sequence. These two paired-up proteins are considered each other’s counterpart protein and only the highest scoring out of the two is retained.

#### Picked Group TDS

This is a target-decoy competition step at the protein group level. First, we mark all leading proteins for each protein group, that is, proteins which cover all identified peptides associated with the protein group. When going down the list of protein groups (sorted by decreasing identification score), protein groups are removed for which one or more counterpart leading protein was observed in the current (higher scoring) protein group. Note that if no protein grouping (nG) is done, this procedure is equal to the pT.

### Protein Group-Level FDR

The null hypothesis for the identification of a protein group (21) was defined as “none of the proteins in the protein group had a correct PSM”, making the alternative hypothesis: “at least one of the proteins in the protein group had at least one correct PSM”. This then leads to the interpretation that if one rejects the null hypothesis, at least one (but potentially multiple or even all) of the proteins in the protein group was correctly identified by a PSM. Note that this hypothesis does not attempt to answer the question of absence and presence of a protein (proteins can be present in the group without having a correct PSM).

To assess which protein groups were correctly identified, we make use of the protein group scores defined above and use the number of decoy protein groups as an estimate for the number of incorrect target protein groups. First, protein groups are sorted by protein group score, starting with the best scoring protein group. The protein group-level FDR for a protein group is estimated as the ratio of the number of decoy protein groups and target protein groups with a better score than the current protein group.

### Summary of Methods

The options listed above for peptide-level FDR threshold, protein grouping, usage of shared peptides, protein scoring, and protein group FDR calculation can be combined in any desired constellation. Below is a summary of the methods used in the main text.

*MaxQuant*

• Grouping: subset grouping (sG)
• Shared peptides: Occam’s razor (rS)
• Scoring: multiplication of MaxQuant PEPs (mmP)
• TDS: classic (cT)

*Savitski*

• Grouping: no grouping (nG)
• Shared peptides: discard (dS)
• Scoring: best Percolator PEP (bpP)
• TDS: picked (pT)

*Picked Protein Group FDR*

• Grouping: rescued subset grouping (rsG)
• Shared peptides: discard (dS)
• Scoring: best Percolator PEP (bpP)
• TDS: picked group (pgT)

*Savitski + Classic FDR*

• Grouping: no grouping (nG)
• Shared peptides: discard (dS)
• Scoring: best Percolator PEP (bpP)
• TDS: classic (cT)

*Discard + Picked Group*

• Grouping: subset grouping (sG)
• Shared peptides: discard (dS)
• Scoring: best Percolator PEP (bpP)
• TDS: picked group (pgT)

*Razor + Picked Group*

• Grouping: subset grouping (sG)
• Shared peptides: Occam’s razor (rS)
• Scoring: best Percolator PEP (bpP)
• TDS: picked group (pgT)

*Classic Protein Group FDR*

• Grouping: rescued subset grouping (rsG)
• Shared peptides: discard (dS)
• Scoring: best Percolator PEP (bpP)
• TDS: classic (cT)

We observed that MaxQuant PSM-level PEPs are less well-calibrated than those generated by Percolator, which led to anticonservative protein group-level FDR estimates for the MaxQuant PEPs but not for the PEPs generated by Percolator (supplemental Fig. S1). Using the Percolator, PEPs had the added benefit of a 7% increase (164k *versus* 154k) in the number of peptide identifications at 1% peptide-level FDR compared to using the MaxQuant PEPs (supplemental Fig. S1*E*). Therefore, the best Percolator PEP (bpP) was selected as the default choice for protein scoring. Results are also shown for mmP and best MaxQuant PEPs where these were relevant.

## Results

### Protein Group-Level FDR Estimation

**Fig. 1:**
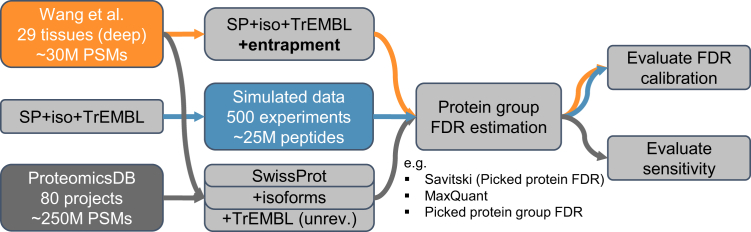
**Overview of datasets and evaluations.** We used entrapment searches on a deep proteome study as well as simulated data to assess the accuracy of FDR estimates of different protein group-level FDR estimation methods. We also evaluated the sensitivity of the methods on three databases with increasing levels of redundancy (SwissProt canonical, SwissProt+isoforms, and SwissProt+iso+TrEMBL) for the deep proteome study and the human section of ProteomicsDB. FDR, false discovery rate.

**Fig. 2:**
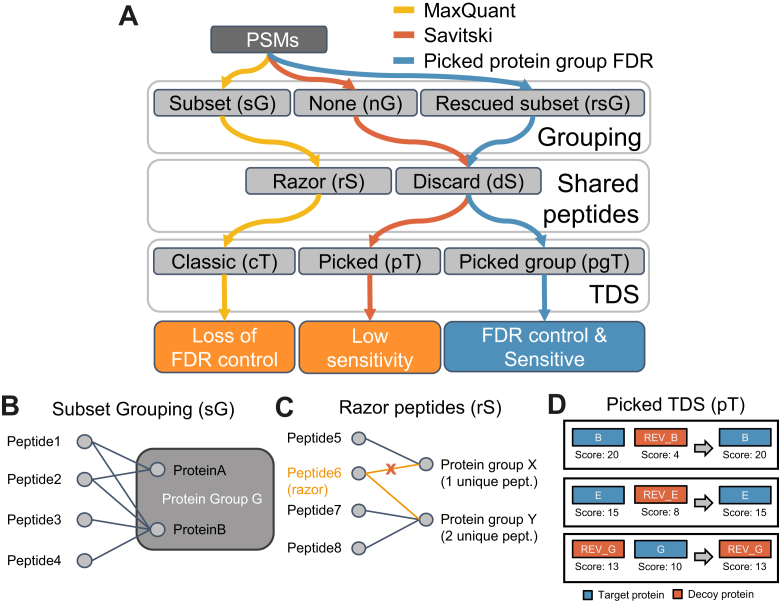
**Overview of protein group-level FDR estimation methods, its constituent stages, and the available options at each stage.***A*, a protein group-level FDR estimation method takes a list of PSMs and chooses one of the available options at each of the three stages: protein grouping, handling of shared peptides, and target-decoy strategy. *B*, subset grouping combines proteins into groups if the peptides of one protein (ProteinA) form a subset of the peptides of another protein (ProteinB). *C*, the razor peptide approach assigns a shared peptide to the protein group with the most unique identifications. *D*, the picked target-decoy strategy performs a target-decoy competition on protein level, retaining only the best scoring protein out of each target-decoy pair. FDR, false discovery rate; PSM, peptide-spectrum match.

Protein group-level FDR estimation can be broken down into multiple stages, where one of several available *options* at each stage has to be chosen. We define a *method* as a particular combination of chosen options. Any one method takes a list of PSMs as input and generates a list of protein groups with associated protein group-level FDRs as output. We used two large-scale datasets to evaluate several protein group-level FDR estimation methods in terms of accuracy and sensitivity (Fig. 1). Specifically, we compare (1) MaxQuant’s method, (2) the Picked Protein FDR method by Savitski *et al.*, and (3) a novel Picked Protein Group FDR method which will be introduced further below. We demonstrate that the two state-of-the-art methods have issues either with calibration or sensitivity and that the Picked Protein Group FDR method resolves both of these problems (Fig. 2*A*).

**Fig. 3:**
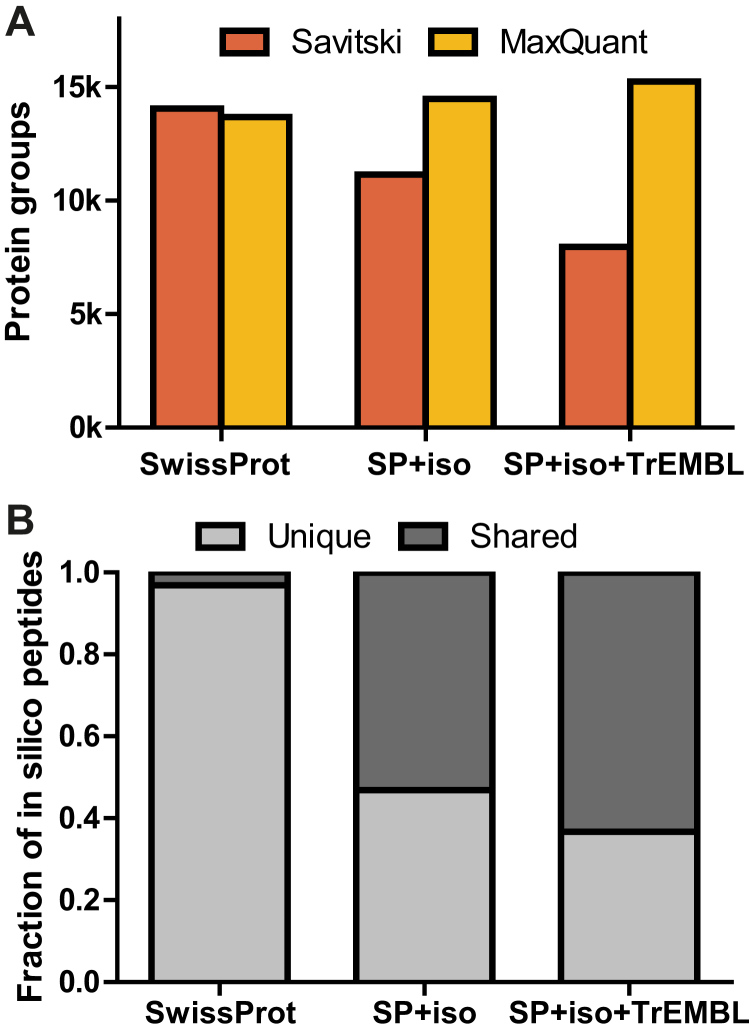
**Shared peptides lead to reduced sensitivity for the Savitski method.***A*, number of identified protein groups at 1% FDR for the Savitski and MaxQuant on the Wang_base dataset. The Savitski method has sensitivity issues for highly redundant databases. *B*, fraction of shared and unique peptides based on in-silico digests of three databases with different levels of redundancy. For databases that include isoforms, the fraction of shared peptides is >50%. FDR, false discovery rate.

The different methods, stages of data processing, and associated options are described in detail in the Experimental Procedures section but a brief overview is given here for convenience. First, one has to decide how proteins are grouped. The simplest option is to not group proteins at all (nG), that is, each protein is its own protein group. sG (Fig. 2*B*) groups proteins if the identified peptides of one protein are a subset of the identified peptides of another protein. Rescued subset protein grouping (rsG) is an extension of sG that will be introduced in more detail further below. Second, peptides shared between protein groups can either be discarded shared peptides (dS) or assigned using Occam’s razor (Fig. 2*C*), that is, assigned to the protein group with the highest number of identified peptides, with ties broken randomly. Next, one has to calculate a score for each protein based on the scores of peptide identifications (omitted from Fig. 2*A* for simplicity). As the default choice, we used bpP among all PSMs for a protein group, while also showing results for mmP and best MaxQuant PEPs where relevant for the comparison of methods. Finally, one has to choose TDS for protein groups. In the classic TDS (cT), all target and decoy protein groups are passed onto the FDR estimation stage. In the pT (Figs. 2 and 3*D*), the score of each target protein is compared to its shuffled or reversed decoy counterpart protein and only the best scoring out of the two proteins is retained. The picked group TDS (pgT) is an evolution of the pT that specifically deals with protein groups and which will also be introduced further below.

As input to the protein group FDR estimation methods, we used the MaxQuant evidence.txt results filtered at 1% peptide-level FDR and 100% protein-level FDR and used Percolator to re-score the PSMs (see Experimental Procedures). All options and methods for protein group-level FDR estimation have been implemented in a Python package named Picked Group FDR (https://pypi.org/project/picked-group-fdr/). The results shown below were generated with v0.3.0.

### The Savitski Method Shows Reduced Sensitivity for Databases Containing Isoforms

The protein FDR estimation method we proposed in Savitski *et al.*, based on the pT, has been shown to avoid the accumulation of decoy matches at the gene level in large datasets, while correctly controlling protein-level FDR (8, 9). We reanalyzed a recently published dataset of deep proteomes of 29 human tissues (30) by searching 50 million tandem mass spectra (MS2) against three human protein sequence databases of different size and containing increasing peptide level sequence redundancy (Swiss-Prot, Swiss-Prot+Isoforms, Swiss-Prot+Isoforms+TrEMBL). This dataset will be referred to as the *Wang_base* dataset.

When only taking canonical protein sequences into account (Swiss-Prot), we observed a 3% gain in identified proteins for the Savitski method compared to MaxQuant’s method (Fig. 3*A*). This modest increase is realized despite the fact that the Savitski method does not perform protein grouping (nG) and dS, whereas MaxQuant’s method uses sG and rS, both of which can enhance sensitivity. When we applied the pT to results of searches against protein databases including isoforms (Swiss-Prot+Isoforms) and unreviewed protein sequences (Swiss-Prot+Isoforms+TrEMBL), the sensitivity of the Savitski method drops compared to MaxQuant’s method (Fig. 3*A*). We also obtained the counter-intuitive result that fewer proteins are identified the larger the database that is used. This drop in sensitivity can largely be attributed to the discarding of the shared peptides in the Savitski method. This is because the fraction of shared peptides increases drastically with the increasing sequence redundancy of tryptic peptides obtained by in-silico digestion (Fig. 3*B*). For the most redundant database, Swiss-Prot+Isoforms+TrEMBL, 63% of all peptides are shared by at least two protein sequences. This effect is also evident in the MaxQuant search results, where 75% of the peptides were shared by two or more protein sequences for this database (supplemental Fig. S3).

### Development of the Picked Protein Group FDR Method

**Fig. 4:**
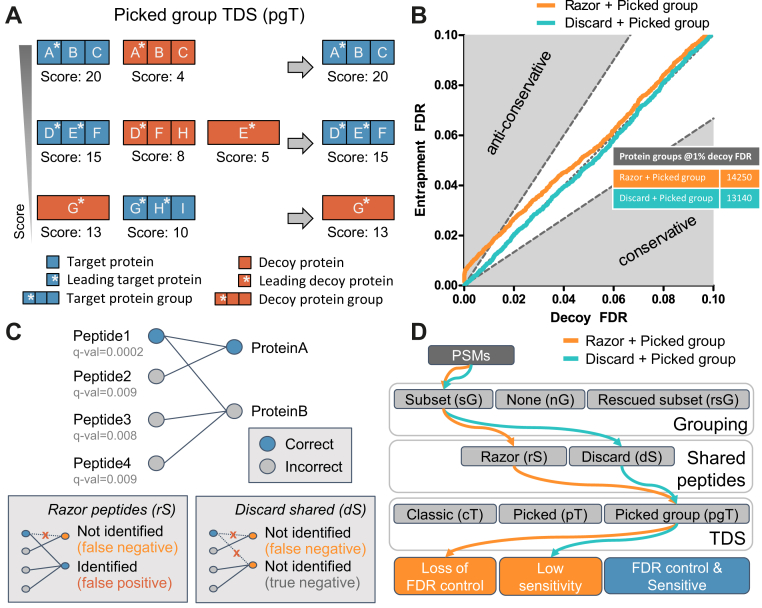
**The picked group target-decoy strategy handles competition of protein groups but does not resolve the issues of the state-of-the-art methods.***A*, the picked group TDS extends the picked TDS to handle protein groups. Protein groups are sorted by decreasing identification score. Going down the sorted list, all groups containing ≥1 counterpart of one of the leading proteins of the current group are eliminated. *B*, protein group-level FDR calibration plots using entrapment searches for two methods using the picked group TDS. The region between y = 1.5× and y = 0.67× (*dashed lines*) was deemed well-calibrated. The Razor + Picked group method produces anticonservative FDR estimates, whereas Discard + Picked group has reduced sensitivity. *C*, the results in panel (*B*) can be explained by this example. Low-confident, incorrect peptides 2-4 prevent proteins A and B from being grouped. Razor peptides (rS) lead to anticonservative estimates due to the erroneous assignment of true positive peptide 1 to the incorrect protein B. If shared peptides are discarded (dS), the high-confident peptide 1 is discarded, leading to reduced sensitivity as neither protein is identified. *D*, schematic summary of the methods evaluated in panel (*B*). FDR, false discovery rate; TDS, target-decoy strategy.

In light of the above, we hypothesized that the negative effect of a high rate of shared peptides on the identification of proteins could be addressed by a more appropriate method of protein grouping and subsequent adjusted FDR estimation. In order to be able to use the pT, we had to solve the issue that target and decoy proteins are not necessarily grouped in a way that would allow fair competition. For example, a target protein group may consist of proteins D, E, and F, whereas the decoy protein group that contains the decoy counterpart of protein D, REV_D, also contains REV_F and REV_H (Fig. 4*A*), whereas REV_E forms a different protein group of its own. Therefore, we extended the pT to handle such cases that arise from protein grouping and we term this extension the pgT. This strategy first sorts the protein groups by descending protein identification score. Then, while going down the sorted protein group list, all protein groups that contain at least one counterpart protein of a leading protein in the current protein group (but with a lower score) are eliminated (Fig. 4*A*, see Experimental Procedures).

To verify that the pgT leads to well-calibrated protein group-level FDRs, we searched the *Wang et al*. dataset against the Swiss-Prot+Isoforms+TrEMBL database augmented with an entrapment database (31) (*Wang_trap_0.5* dataset, see Methods). This entrapment database was constructed in such a way that it mimicked the proportion of shared peptides as found in the Swiss-Prot+Isoforms database (50% shared peptide ratio). When using pgT together with sG and dS, we observed that the resulting FDRs were well-calibrated over the entire FDR range (Fig. 4*B*). However, when we changed this method to use rS, the results showed anticonservative FDR estimates. A similar behavior was observed for the MaxQuant method, because it also uses rS.

The anticonservative behavior when using rS is a result of false positives that remain unaccounted for by the decoy model, as explained in the introduction and in Figure 4*C*. This effect becomes stronger as more false positives are included in the input list of PSMs, for example, when applied to large-scale datasets or when permissive PSM-level FDR cutoffs are used. Neither using different scoring methods nor the pgT was able to lead to well-calibrated protein group-level FDRs when using rS (supplemental Fig. S4). When reducing the shared peptide ratio to 4% (supplemental Fig. S5; *Wang_trap_0.04* dataset, mimicking Swiss-Prot’s shared peptide ratio), this effect was not as apparent anymore, as the effect of rS is reduced.

Combining pgT with dS led to well-calibrated FDR estimates but the loss in the number of identified protein groups was not completely resolved (Fig. 4*B*). This is because a group of proteins with one (or more) high-confident shared peptide identifications can be split over two protein groups owing to the presence of low-confident peptides that are unique to particular isoforms and are present in the same group (Fig. 4*C*). In such cases, high-confidence shared peptides are shared by two protein groups and are discarded by pgT. This results in neither protein group being identified, in turn, leading to reduced sensitivity of protein group identification.

**Fig. 5:**
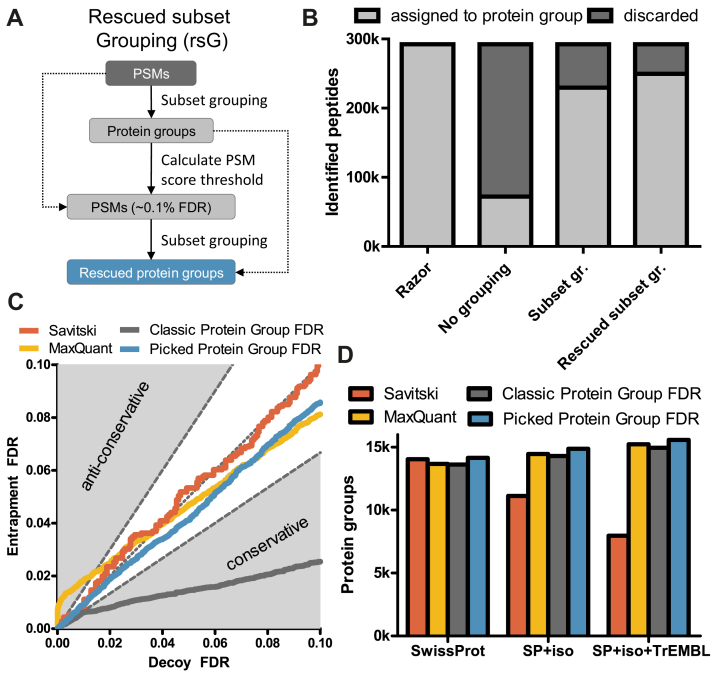
**The Picked Protein Group FDR method is sensitive and well-calibrated.***A*, rescued subset grouping employs a second protein grouping that prevents low-scoring false positives from breaking up protein groups. *B*, Number of identified peptides assigned to a protein group or discarded for the different protein grouping options for Wang_base searched against the SP+iso+TrEMBL database. Rescued subset grouping further improves the number of assigned peptides to protein groups compared to subset grouping. *C*, protein group-level FDR calibration plots using entrapment searches. The Picked Protein Group FDR method shows good calibration, whereas MaxQuant method produces anticonservative FDR estimates. *D*, bar plots of the number of identified protein groups at 1% FDR. The Picked Protein Group FDR method consistently identifies the most protein groups across the three databases with different levels of redundancy. FDR, false discovery rate.

To overcome the shortcomings of (1) using rS, leading to anticonservative protein FDR estimates (Fig. 4*D*, orange), and (2) dS, leading to reduced sensitivity (Fig. 4*D*, purple), we propose an extension to sG that we term rsG (Fig. 5*A*). First, a regular sG is performed and the PSM-level score cutoff corresponding to a 1% protein group-level FDR is computed. Second, using this cutoff, the high-confident PSMs are retained from the original list and sG is performed on this filtered list of PSMs. The final list of protein groups consists of the protein groups from the second grouping, supplemented with protein groups from the first grouping for which none of its proteins were already in a protein group from the second grouping. By removing the effect of low-confident PSMs on the protein grouping procedure, more high-confidence peptides can be uniquely mapped to a protein group. This reduces the fraction of discarded precursors from 0.21 for sG to 0.15 for rsG, a 30% decrease (Fig. 5*B*). On the *Wang_trap_0.5* dataset, pgTDS combined with dS and rsG (rsG, dS, bpP, pgT) shows well-calibrated FDRs (Fig. 5*C*). This combination of options in this new method will henceforth be referred to as the *Picked Protein Group FDR method*.

We analyzed the *Wang_base* dataset using the new Picked Protein Group FDR method and compared the results to those obtained by MaxQuant’s method (sG, rS, mmP, cT), the Savitski method (nG, dS, bpP, pT), and rsG with the cT (bpP, rsG, dS, cT). For the database with the smallest ratio of shared peptides (Swiss-Prot), the pT of the Savitski method led to the expected moderate increase in identified proteins at 1% protein group-level FDR compared to MaxQuant’s method which uses the cT. The same was observed for the Picked Protein Group FDR method, which showed an increase of 4% in the number of identified protein groups relative to MaxQuant’s method. When including isoforms into the analysis (Swiss-Prot+Isoforms), the number of identified proteins using the Savitski method drops by 21%, as observed above. In contrast, this is not the case for methods that use rsG, for which an increase of 4 to 5% in the number of identified protein groups was observed compared to the Swiss-Prot database. This is because one can now identify multiple protein groups per gene. For the Swiss-Prot+Isoforms+TrEMBL database, the number of protein groups at 1% FDR roughly doubled when using the Picked Protein Group FDR method compared to the Savitski method (Fig. 5*D*). There was also an increase of 4% in the number of identified protein groups by switching from cT to pgT and a 2% increase compared to MaxQuant’s method. However, as demonstrated above, the FDR estimates of MaxQuant’s method are likely not well-calibrated due to the use of rS. Hence, the actual FDR might be higher than the reported 1% on this list of protein groups. In summary, the Picked Protein Group FDR method obtained the highest number of identified protein groups across the three differently sized databases while correctly controlling protein group-level FDR. This led to 15,600 confidently identified protein groups in this human proteome represented by 29 healthy tissues.

### The Picked Protein Group FDR Method Scales to Very Large Datasets

Next, we evaluated if the Picked Protein Group FDR method would scale to analyzing very large datasets. Because entrapment database searches are computationally expensive when performed at scale and because they change the original database in terms of size and shared peptides, we instead used simulated data (see Experimental Procedures) as a way to generate large-scale datasets to verify protein group-level FDR estimates. To verify the validity of the simulated data, we checked if we could recover the qualitative effects observed for the *Wang_trap_0.5* and *Wang_trap_0.04* datasets. To this end, we estimated the appropriate input parameters for the simulation from the entrapment experiments using the Wang *et al.* dataset. We then simulated PSMs for the two entrapment searches (4% and 50% shared peptide ratios), where each experiment was controlled at 10% or 1% peptide-level FDR. The results of the different protein group FDR methods for simulated and entrapment datasets were similar for both shared peptide ratios (supplemental Fig. S6). For example, we observed the aforementioned anticonservative behavior resulting from allowing rS in the 50% shared peptide ratio data which was hardly noticeable at the 4% shared peptide ratio.

**Fig. 6:**
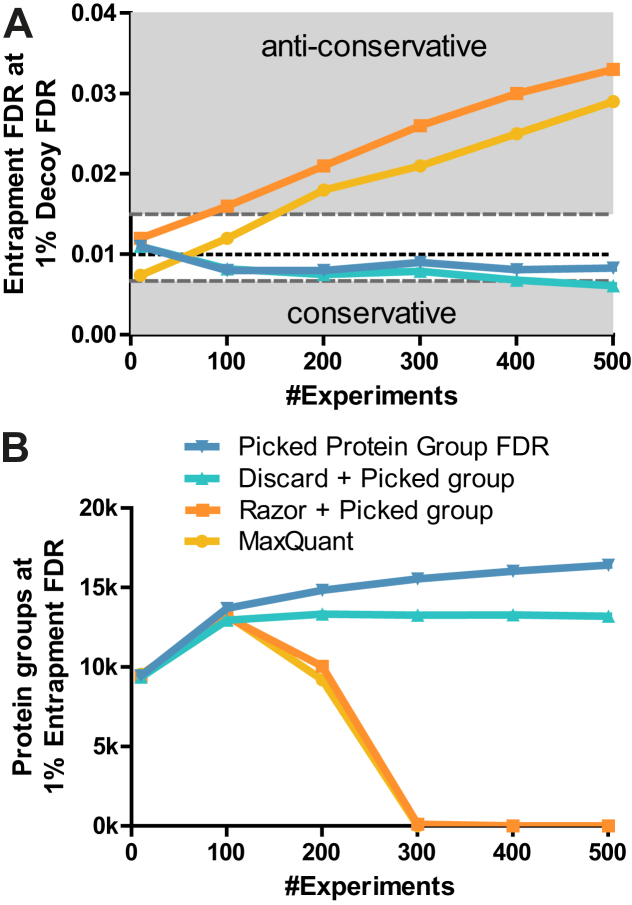
**The Picked Protein Group FDR method performs well when combining hundreds of simulated experiments in a single analysis.***A*, protein group-level FDR calibration plot showing the entrapment FDR at 1% decoy FDR for 10 to 500 combined experiments with ∼10,000 proteins each. The Picked Protein Group FDR produces accurate FDR estimations regardless of the number of combined experiments. The two methods using razor peptides (MaxQuant and Razor+Picked group) produce increasingly anticonservative estimates as more experiments are combined. *B*, the number of protein groups at 1% entrapment FDR for different numbers of combined experiments. As desired, the Picked Protein Group FDR method increases the number of identified protein groups as more experiments are combined. Above 300 experiments, methods using razor peptides have too many high-scoring entrapment protein groups such that no protein groups make the 1% entrapment FDR. FDR, false discovery rate.

We next simulated data to investigate the effect of combining hundreds of experiments containing about 10,000 proteins each and searched against Swiss-Prot+Isoforms+TrEMBL and controlled at 1% peptide-level FDR per experiment (Fig. 6 and supplemental Fig. S7). As expected, the more experiments were combined, the larger the anticonservative effect of rS became (Fig. 6*A*). At the same time, the number of protein groups at 1% FDR for the Picked Protein Group FDR method increased as the number of combined experiments increased (Fig. 6*B*). Furthermore, and as expected, using a 10% peptide-level FDR threshold per experiment greatly exacerbated the anticonservative behavior of methods employing rS (supplemental Fig. S8). Reassuringly, the current best practice in proteomics of applying a global 1% peptide-level FDR after combining all experiments did largely resolve the calibration issues caused by rS, although some anticonservative behavior could still be observed in the very low FDR region. However, this did not lead to more identified protein groups compared to the Picked Protein Group FDR method (supplemental Fig. S9).

**Fig. 7:**
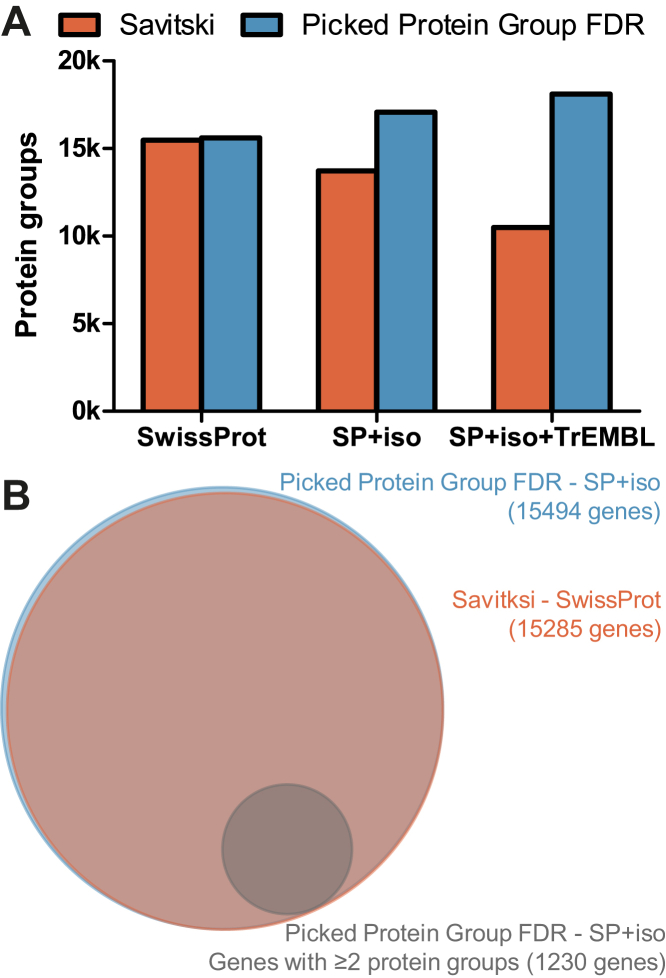
**Reanalysis of the human section of ProteomicsDB with the Picked Protein Group FDR method leads to increased information for databases including isoforms.***A*, bar plot showing the number of protein groups at 1% FDR. The Picked Protein Group FDR method exhibits an increase in the number of protein groups as isoforms and unreviewed proteins are included in the database. This is mainly because multiple protein groups can now be identified per gene. *B*, Venn diagram on gene level comparing the Savitski method without isoforms (*red*) and the Picked Protein Group FDR method with isoforms (*blue*). The Picked Protein Group FDR method reveals information about protein isoforms, with 8% of the identified genes having multiple identified protein groups (*gray*). FDR, false discovery rate.

Finally, we applied the Picked Protein Group FDR method to the reanalysis of the entire human section of ProteomicsDB which comprises 77 projects representing many different types of proteomic applications, 19,800 LC-MS/MS runs leading to 410 million PSMs. As expected, we observed a sharp increase of 71% in the number of identified protein groups compared to the Savitski method (18,000 *versus* 10,500) when searching against Swiss-Prot+Isoforms+TrEMBL but identified an almost identical number when searching the canonical Swiss-Prot database only (15,600 *versus* 15,500; Fig. 7*A*). Furthermore, the Picked Protein Group FDR method showed the expected behavior of identifying more protein groups when searching Swiss-Prot+Isoforms+TrEMBL (18,000) than Swiss-Prot+isoforms (17,000) or Swiss-Prot (15,600). At the gene level, we observed the expected and desired behavior that the same genes were identified when searching Swiss-Prot+Isoforms or Swiss-Prot (Fig. 7*B*). Searching Swiss-Prot+Isoforms, the Picked Protein Group FDR method resulted in 1230 genes with multiple identified protein groups (supplemental Table S2).

## Discussion

Here, we introduced the Picked Protein Group FDR method for calculating protein group-level FDRs. This method achieves higher sensitivity than alternative state-of-the-art methods, correctly controls protein group-level FDR, and scales well to repository-sized datasets. We developed a Python package and an accompanying graphical user interface that implements this method. This software tool can directly be applied to MaxQuant search results, with the option of combining multiple search results in a single protein group analysis. On a deep proteome study of 29 healthy human tissues (Wang_base), the number of identified protein groups was increased by up to 500 (+4%) compared to MaxQuant’s method. The reanalysis of the human section of ProteomicsDB resulted in 15,600 identified genes, similar to the number previously obtained with the Savitski method. Out of these identified genes, 1230 had multiple identified protein groups when searched against the SwissProt database with isoforms included. This number is substantially higher than the 246 genes previously reported by Abascal *et al.* (32). These authors purposely employed very stringent selection criteria in order to minimize the number of false positives, as the authors had observed artifacts of unaccounted false positives on peptide level (33). Here, we used a 1% protein group-level FDR filter, which corresponded to a rather stringent 0.12% peptide-level FDR filter. Manual inspection of the results indicates that in the vast majority of such genes, high-confident peptides are available unique to each of the protein groups. However, further research will be needed to assess the validity of these results.

To resolve sensitivity and calibration issues of the state-of-the-art methods, the Picked Protein Group FDR method introduces rsG for protein grouping and pgT for target-decoy competition of protein groups. rsG extends regular subset grouping with a second protein grouping step in which low-confident PSMs are ignored. This is comparable to the current best practice of applying a global 1% peptide-level FDR cutoff before protein grouping but has the benefit of producing FDR estimates for protein groups without peptides below the peptide-level FDR cutoff. The pgT extends the pT to handle protein groups. It is easy to implement and is identical to the pT when protein grouping is not performed. However, it should be noted that the pgT is a heuristic rule that relies on the similarity in composition, for example, number of proteins and shared peptides, of incorrect target protein groups and decoy protein groups. One can indeed construct (artificial) examples where pgT produces undesirable results, for example, an incorrect target protein group consisting of one protein eliminating a decoy protein group with 10 decoy proteins that happen to share one peptide. Nevertheless, we demonstrated here through our calibration experiments that, in practice, the protein groups competing against each other are similar enough to ensure a fair competition and, thereby, accurate FDR estimations.

Furthermore, we demonstrated that the use of rS can lead to anticonservative protein group-level FDR estimates. Fortunately, we observed in our simulation experiments that this bias will likely be minor if the best practice of applying a global 1% peptide-level FDR cutoff before protein grouping is used. We acknowledge that rS increase the number of peptides for a protein group and thereby stabilize protein abundance estimates. However, it cannot be guaranteed that the assignment to one of the protein groups in question is indeed correct. Using rS might, therefore, lead to a false sense of confidence in the presence and abundance of specific isoforms.

More concerningly for protein quantification, isoform-specific peptides are frequently only identified in a small fraction of samples. This often leads to high levels of missing values for isoforms, regardless of whether rS are used or not. One way to address this issue could be to take the abundances of the peptides shared between isoforms into account (34, 35). As such methods still have to prove their reliability, we recommend doing differential abundance analysis on gene level and using isoform-level quantification only in cases where enough information is available.

In summary, the current study presents a method for protein group FDR estimation that is both correct and sensitive. The accompanying software as well as the data simulation scripts are open-source, providing the proteomics community useful new tools to design, develop, and test methods for estimating protein group-level FDRs. The authors also expect that the ability of the software to generate consistent protein group identifications when combining search results from different (and possibly large) datasets will make proteomic experiments more comparable without the need for expending large computational resources.

## Data availability

Raw files for the Wang *et al.* dataset are available on PRIDE (PXD010154). The MaxQuant search results and result files of the Picked Protein Group FDR analysis are available on Zenodo (10.5281/zenodo.7157677). The software and graphical user interface are freely available on GitHub (https://github.com/kusterlab/picked_group_fdr) and as a Python package (https://pypi.org/project/picked-group-fdr/). This includes the scripts to reproduce the figures in this article as well as those for simulating PSMs for large-scale datasets.

## Supplemental data

This article contains supplemental data.

## Conflict of interest

M. W. and B. K. are founders and shareholders of OmicScouts GmbH and MSAID GmbH, both operating in the field of proteomics. They have no operational role in either company. All other authors declare that they have no conflicts of interest with the contents of this article.
