# Supplementary material for: Reanalysis of ProteomicsDB Using an Accurate, Sensitive, and Scalable False Discovery Rate Estimation Approach for Protein Groups
Source: Mol Cell Proteomics. 2022 Nov 1;21(12):100437. doi: 10.1016/j.mcpro.2022.100437 (PMC9718969; doi:10.1016/j.mcpro.2022.100437)
Supplement: Supplemental Figures S1–S9 [file mmc1.pdf]

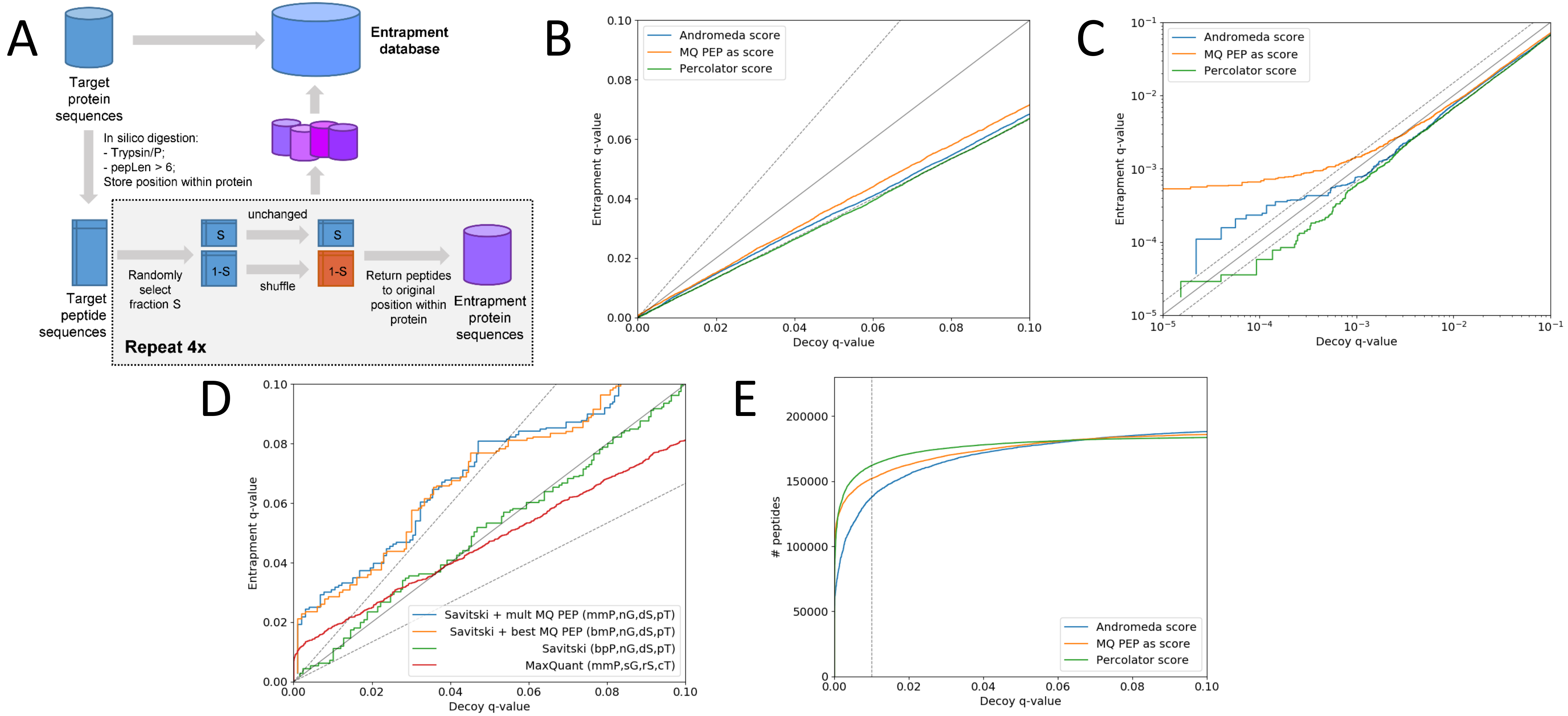

Suppl. Fig. 1: (A) Schematic overview of the creation of the entrapment database. (B) Peptide-level FDR calibration plots based on entrapment searches for three PSM scoring methods. The Andromeda, MaxQuant PEP and Percolator scores are all well-calibrated (though conservative) at high FDR values. (C) same as in (B) but now plotted in log scale. Both MaxQuant-based scores show an anti-conservative bias for very low q-values. (D) Protein group-level FDR calibration plots based on entrapment searches. The anti-conservative bias observed for both MaxQuant-based PSM scores in (C) creates problems for protein-level scores because protein scores mostly depend on the low q-value tail of the peptide score distribution. (E) Number of identified peptides as a function of the peptide-level FDR.

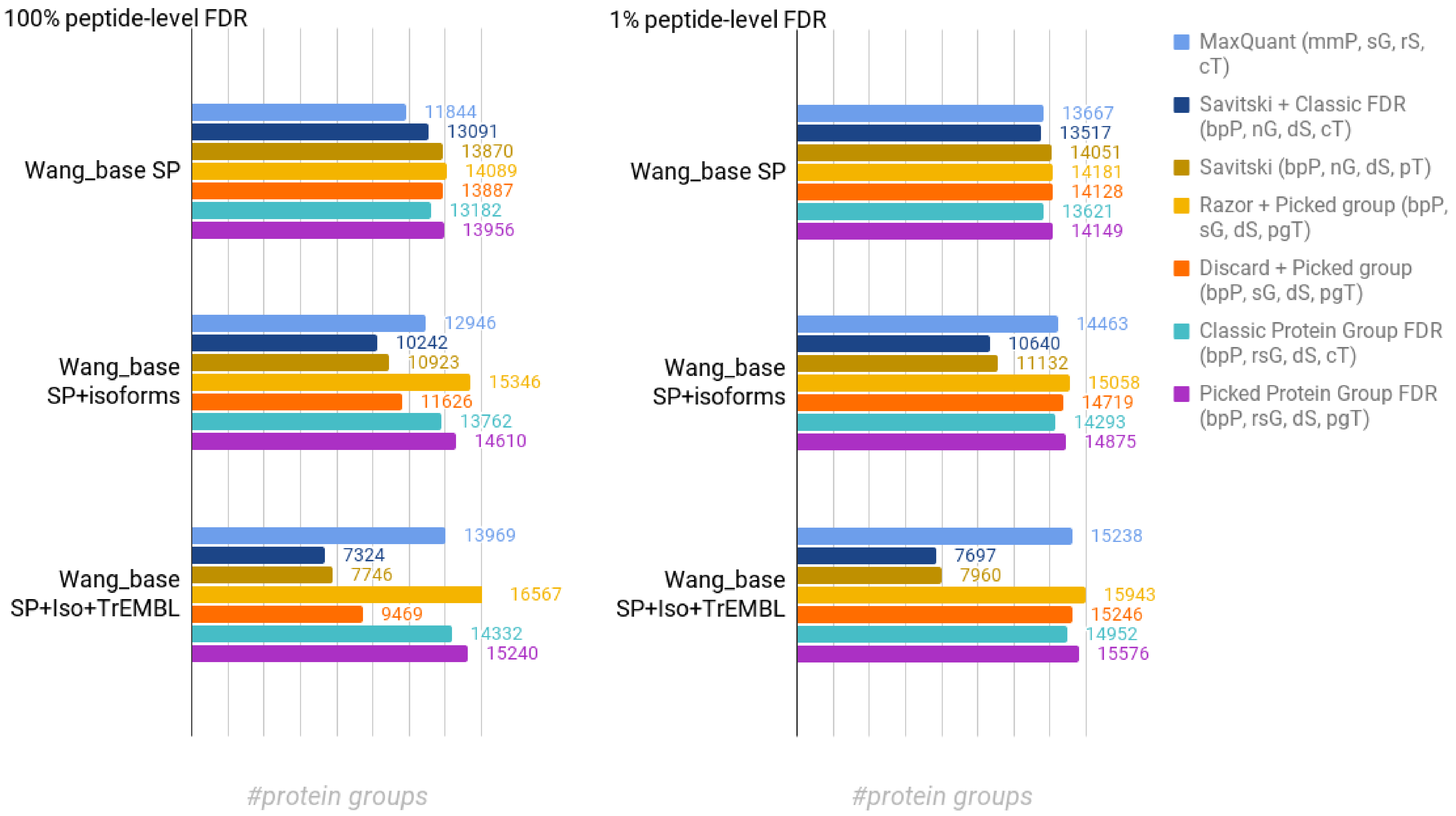

Suppl. Fig. 2: Number of identified protein groups at 1% FDR for different methods for the Wang_base dataset. Note that we demonstrate in the main text that the MaxQuant and Razor + Picked group protein group FDRs are not well-calibrated and likely underestimate the true number of false positives.

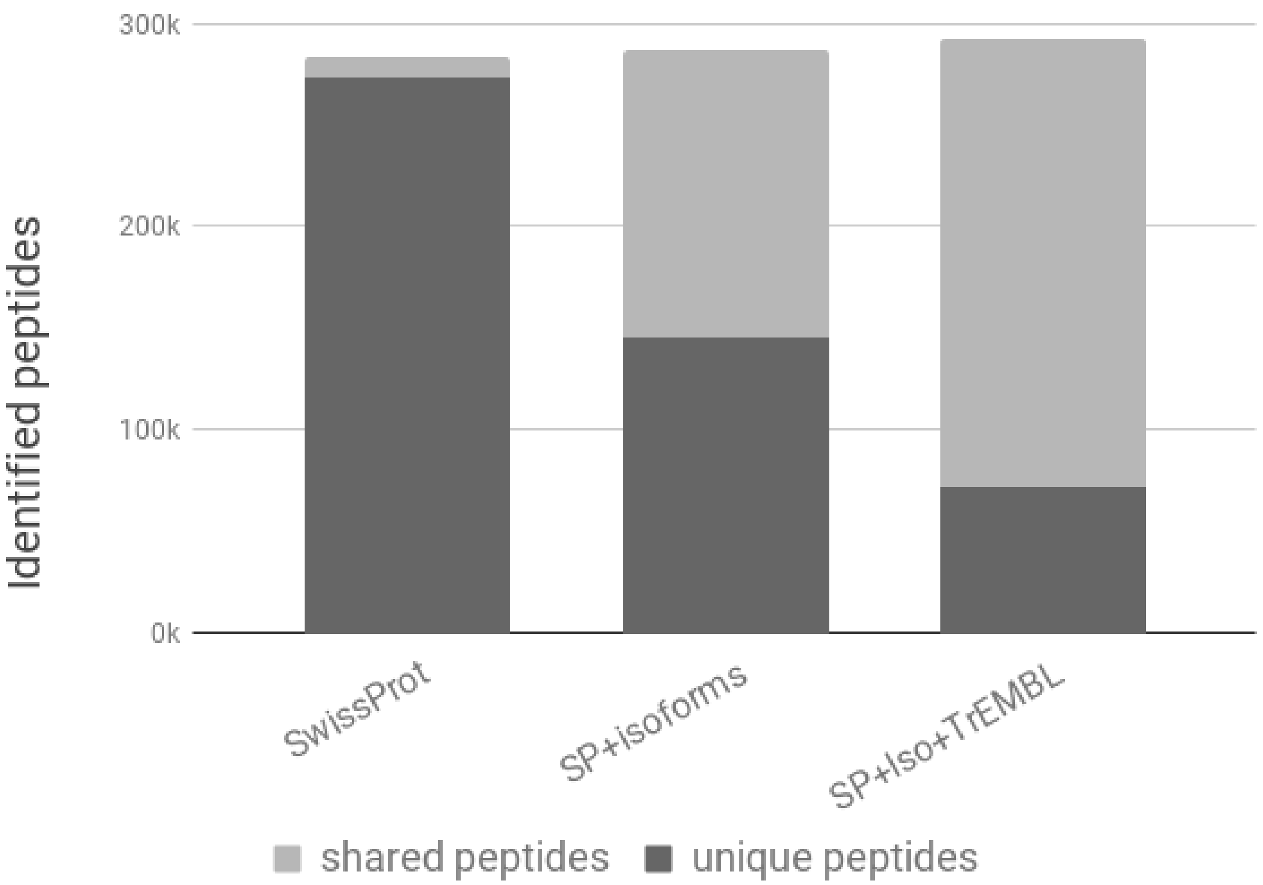

Suppl. Fig. 3: Number of shared and unique peptides in for the Wang_base dataset for three databases with different levels of redundancy. We see a modest increase in total number of peptides with increasing database size, owing to the extra (often highly redundant) protein sequences. More importantly, we see that the percentage of unique peptides drops from 97% (SwissProt) to just 25% (SP+Iso+TrEMBL).

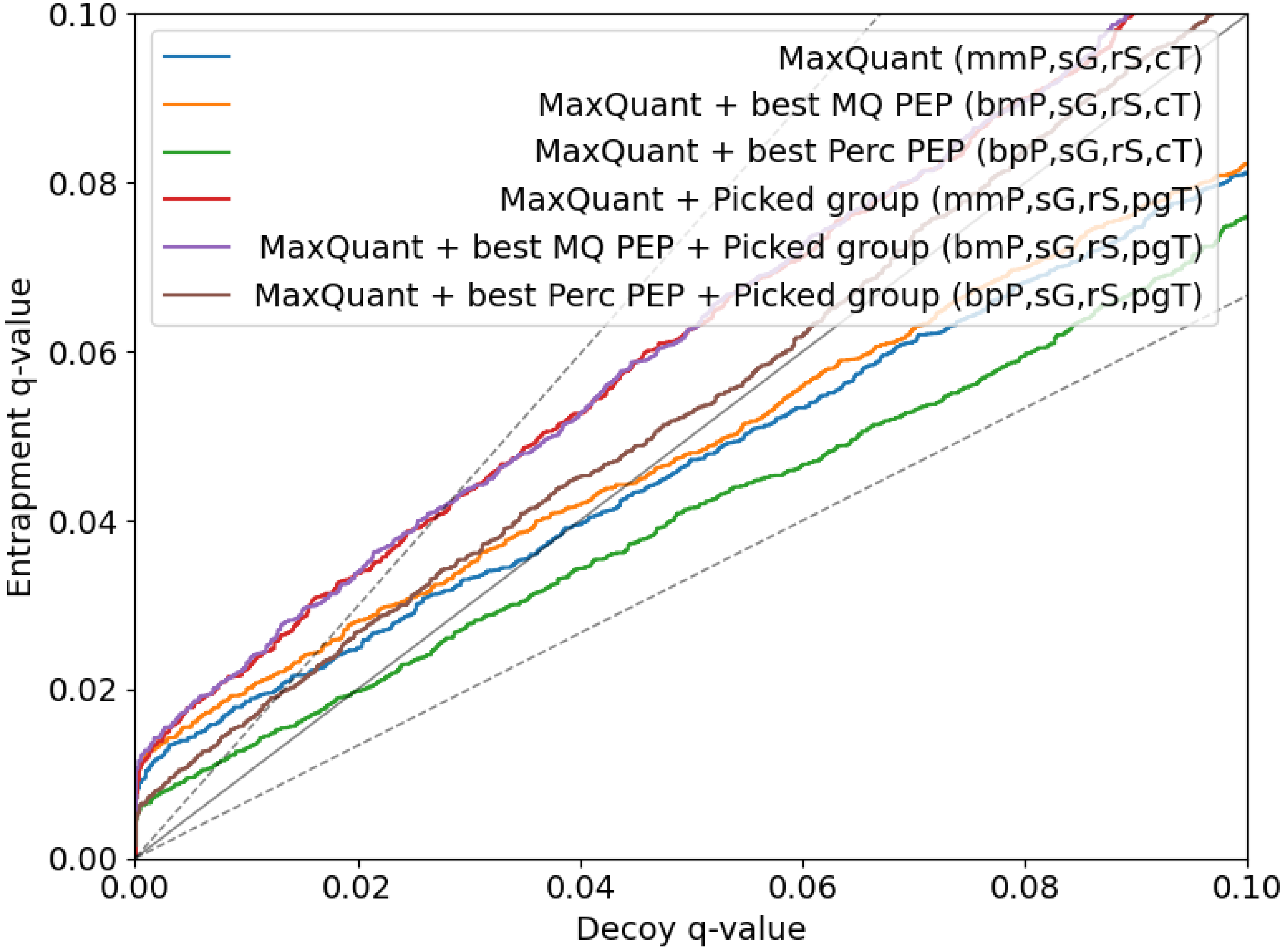

Suppl. Fig. 4: Protein group-level FDR calibration plots using entrapment searches. None of the tested methods that include razor peptides show well-calibrated FDR estimates.

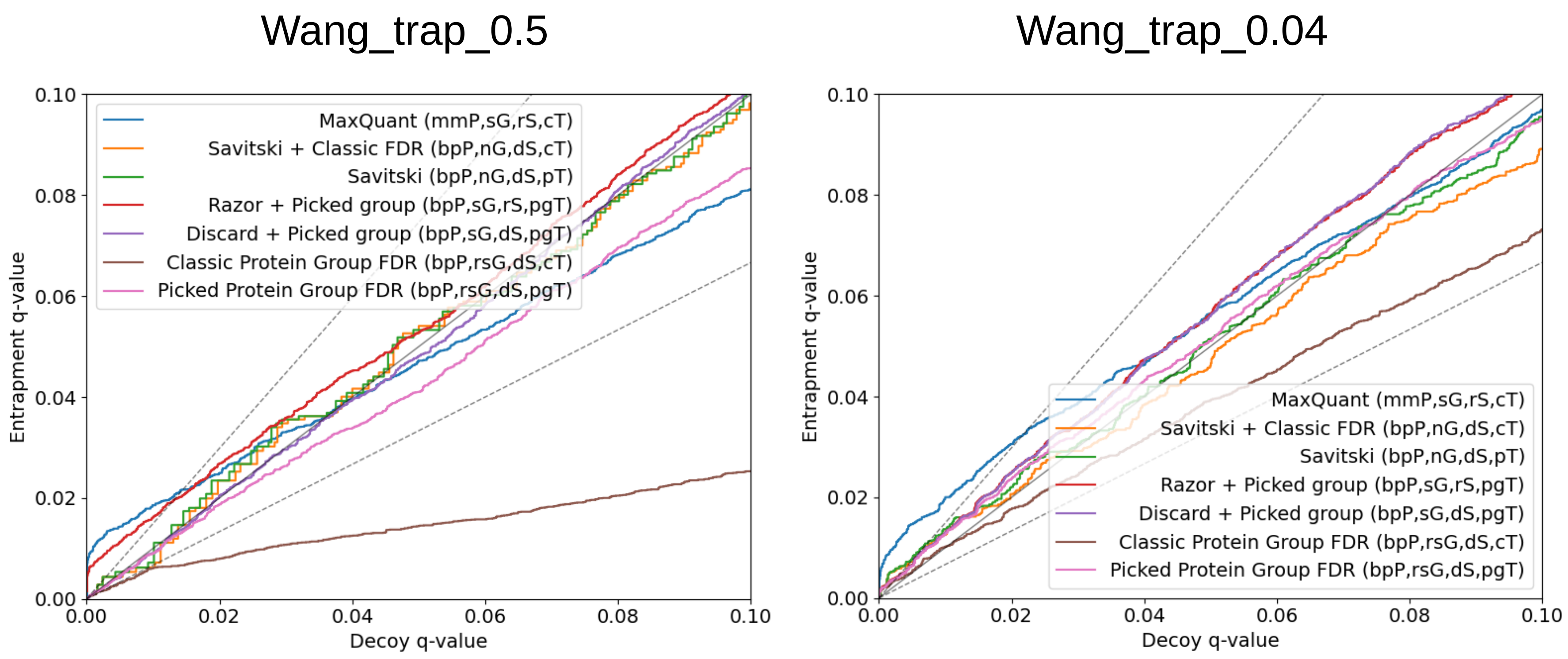

Suppl. Fig. 5: Protein group-level FDR calibration plots using entrapment searches. The poor calibration due to the usage of razor peptides is more apparent in databases with a high rate of shared peptides (left, simulated shared peptide rate of 50%, comparable to SP+TrEMBL) than at low rates (right, simulated shared peptide rate of 4%, comparable to canonical SwissProt).

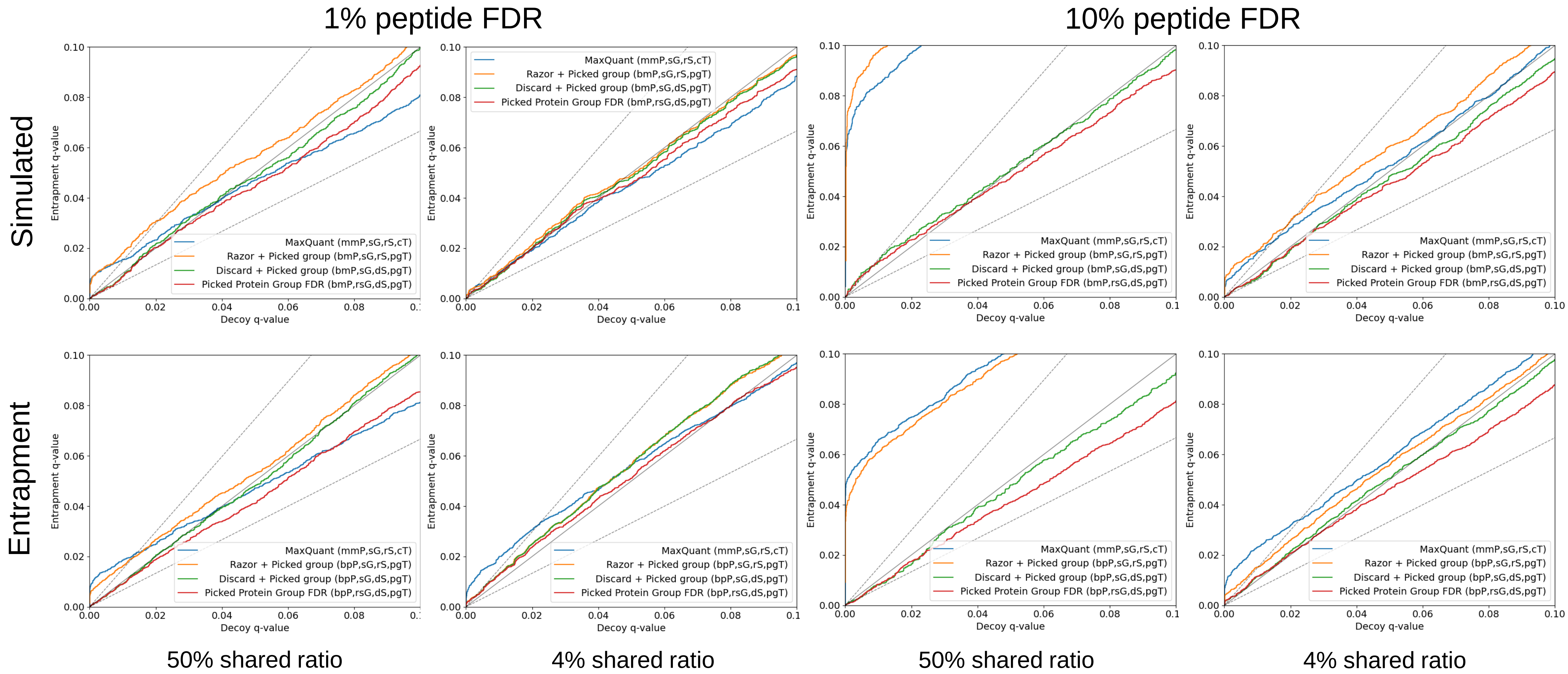

Suppl. Fig. 6: Protein group-level FDR calibration plots using simulated data (bottom) and entrapment searches (top) for 1% (left) and 10% (right) peptide-level FDR and 50% and 4% shared peptide ratio. The results on our simulated data show good qualitative correspondence to our results on entrapment databases. Particularly, it shows the poor calibration of methods with razor peptides on a entrapment database with 50% shared peptide ratio, which largely disappears at 4% shared peptide ratio.

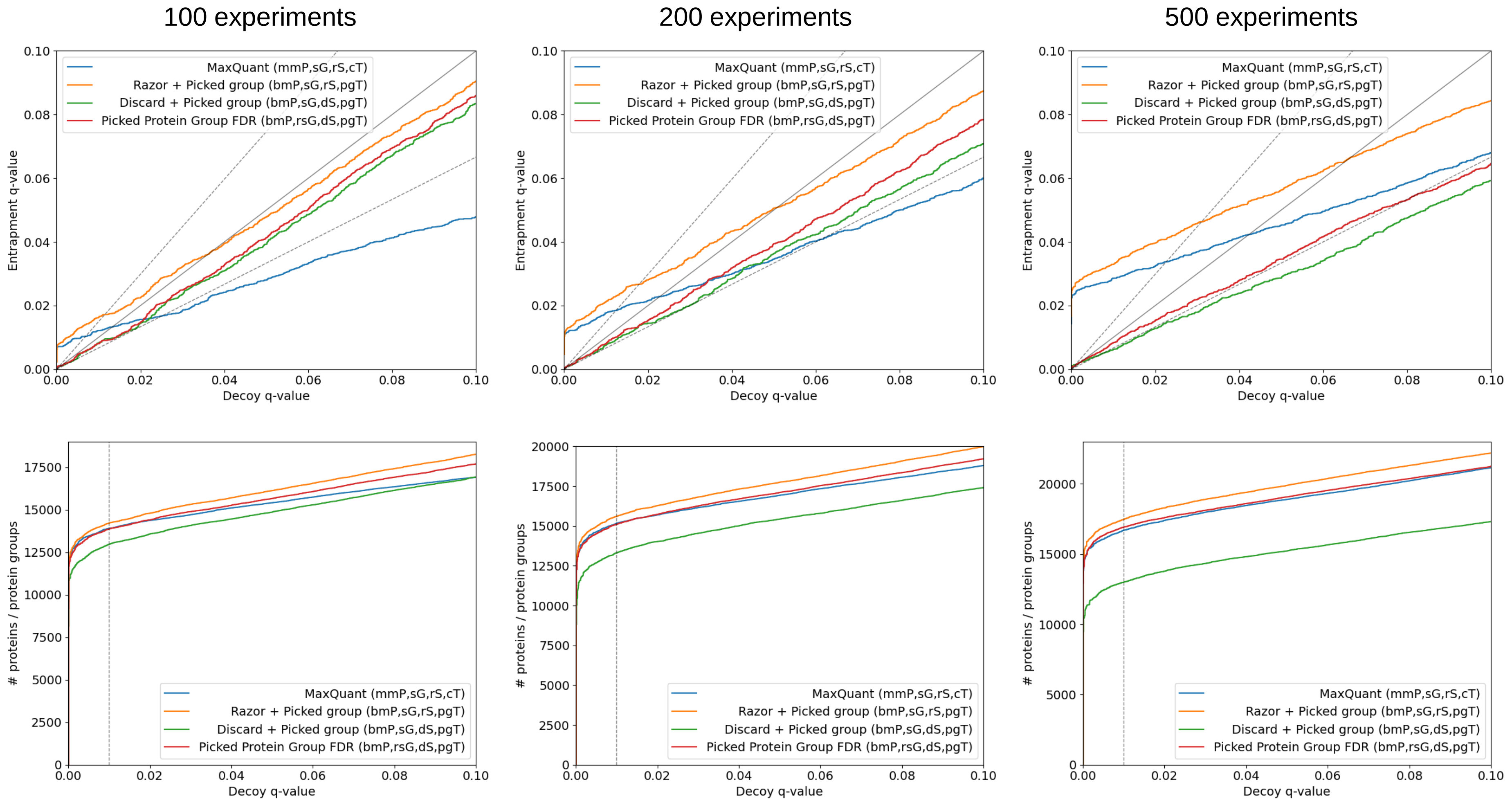

Suppl. Fig.7: Protein group-level FDR calibration plots (top) and number of identified protein groups vs decoy FDR (bottom) for 100, 200 and 500 simulated experiments combined in a single analysis. Each simulated experiment was filtered at 1% peptide-level FDR. We observe the good calibration of the Picked Protein Group FDR method and the increasing issues with methods that use razor peptides as more experiments are combined.

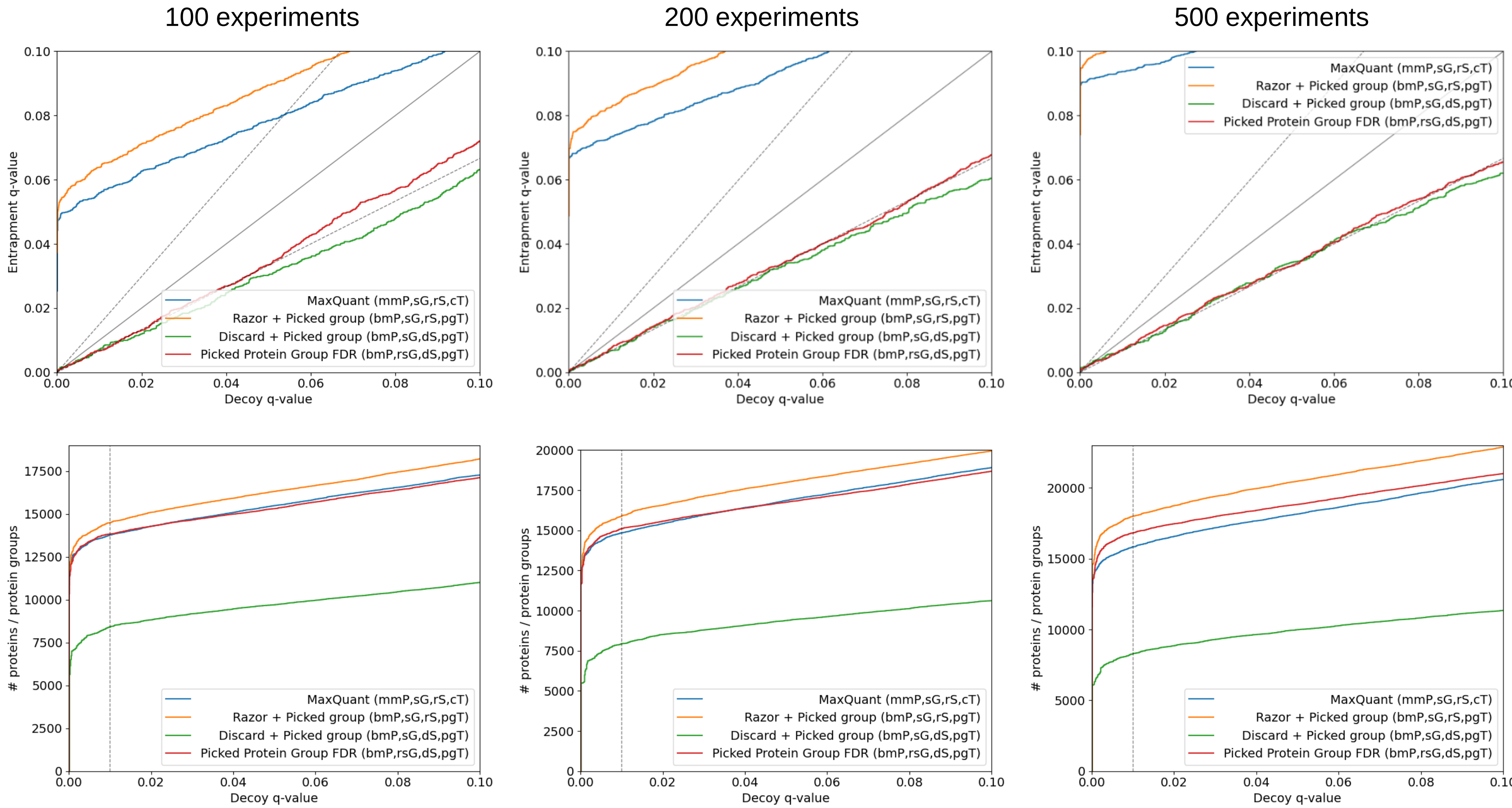

Suppl. Fig. 8: Protein group-level FDR calibration plots (top) and number of identified protein groups vs decoy FDR (bottom) for 100, 200 and 500 simulated experiments combined in a single analysis. Each simulated experiment was filtered at 10% peptide-level FDR. This results in large problems with FDR calibration for methods that use razor peptides, but shows good calibration for our Picked Protein Group FDR method.

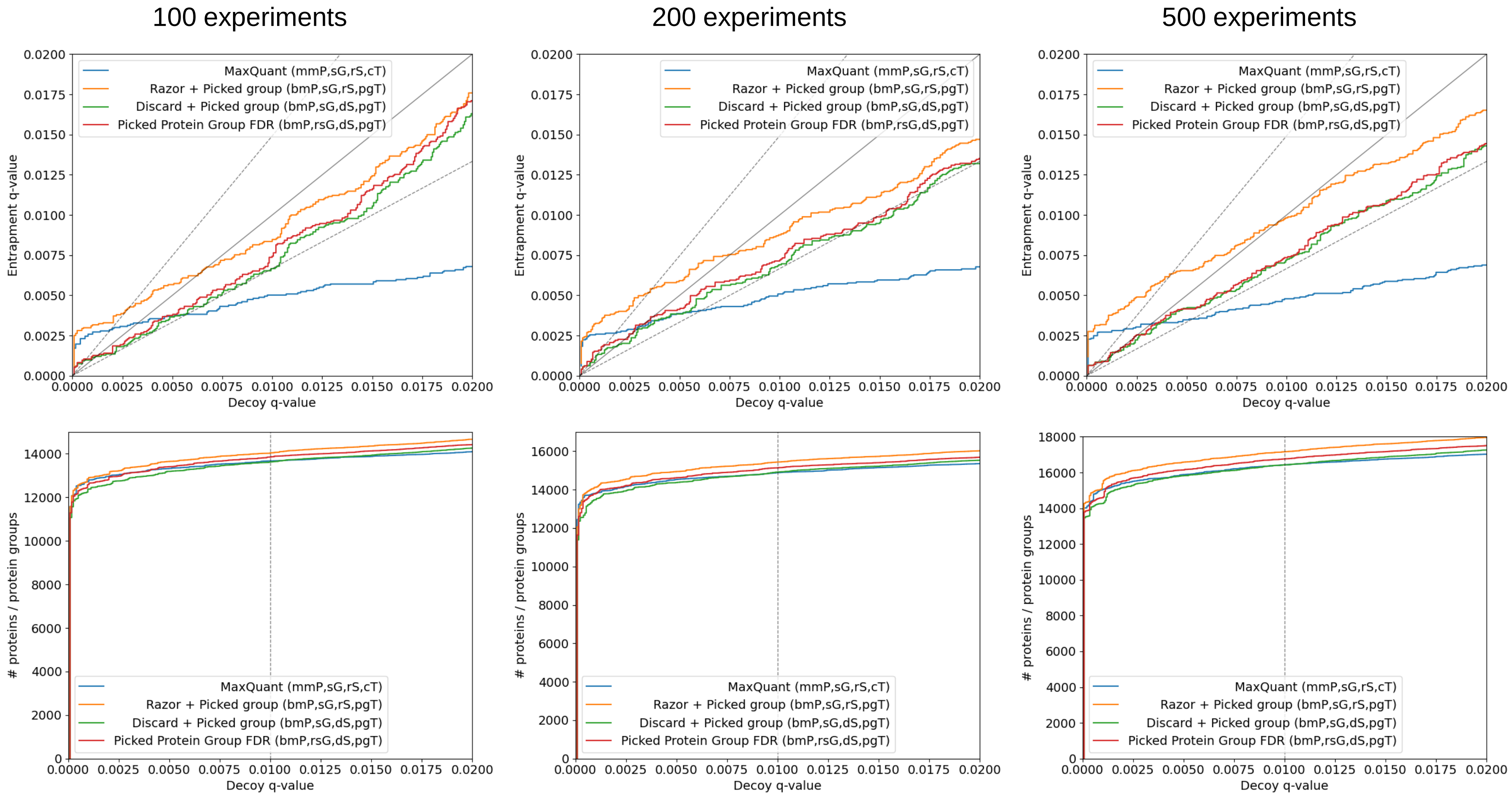

Suppl. Fig. 9: Protein group-level FDR calibration plots (top) and number of identified protein groups vs decoy FDR (bottom) for 100, 200 and 500 simulated experiments combined in a single analysis. After merging all simulated experiments, a global 1% peptide-level FDR cutoff was applied. The FDR bias stays within reasonable bounds for all methods at 1% decoy FDR. However, as more experiments are combined, the anti-conservative bias at low FDRs of methods with razor peptides becomes more apparent.
